# Development of an efficient RNA interference method by feeding for the microcrustacean *Daphnia*

**DOI:** 10.1186/s12896-015-0209-x

**Published:** 2015-10-07

**Authors:** Charles A. Schumpert, Jeffry L. Dudycha, Rekha C. Patel

**Affiliations:** Department of Biological Sciences, University of South Carolina, 700 Sumter Street, Columbia, SC 29208 USA

**Keywords:** RNA interference, *Daphnia*, Knockdown, Feeding, Melanin, *Phenoloxidase*

## Abstract

**Background:**

RNA interference (RNAi) is an important molecular tool for analysis of gene function *in vivo. Daphnia*, a freshwater microcrustacean, is an emerging model organism for studying cellular and molecular processes involved in aging, development, and ecotoxicology especially in the context of environmental variation. However, in spite of the availability of a fully sequenced genome of *Daphnia pulex*, meaningful mechanistic studies have been hampered by a lack of molecular techniques to alter gene expression. A microinjection method for gene knockdown by RNAi has been described but the need for highly specialized equipment as well as technical expertise limits the wider application of this technique. In addition to being expensive and technically challenging, microinjections can only target genes expressed during embryonic stages, thus making it difficult to achieve effective RNAi in adult organisms.

**Results:**

In our present study we present a bacterial feeding method for RNAi in *Daphnia*. We used a melanic *Daphnia* species (*Daphnia melanica*) that exhibits dark pigmentation to target phenoloxidase, a key enzyme in the biosynthesis of melanin. We demonstrate that our RNAi method results in a striking phenotype and that the *phenoloxidase* mRNA expression and melanin content, as well as survival following UV insults, are diminished as a result of RNAi*.*

**Conclusions:**

Overall, our results establish a new method for RNAi in *Daphnia* that significantly advances further use of *Daphnia* as a model organism for functional genomics studies. The method we describe is relatively simple and widely applicable for knockdown of a variety of genes in adult organisms.

## Background

In order to study gene function in intact organisms, effective techniques to manipulate gene expression to achieve an over-expression or knockdown are essential. RNA interference (RNAi) has revolutionized several fields of biology by making it possible to study loss-of-function effects in various organisms without the time-consuming, laborious genetic manipulations. RNAi is a mechanism in which dsRNA molecules trigger gene silencing in a sequence-specific manner, usually resulting in degradation of the transcript complementary to one strand of the dsRNA ([1] and the references within). The RNAi pathway is conserved throughout eukaryotes with examples of the RNAi mechanism being used to silence gene expression in numerous model organisms including (but not limiting to) *Schizosaccharomyces pombe*, *Tetrahymena*, *Drosophila melanogaster*, *Caenorhabditis elegans*, *Danio rerio*, *Xenopus*, and *Mus musculus* [2]. This conserved mechanism of gene silencing has led to exceptional use of reverse genetics methods and has led to a better understanding of molecular pathways at mechanistic levels [3]. Beyond enabling the study of diminished expression of a particular gene leading to advancements in understanding molecular pathways, RNAi has demonstrated a potential for being used in therapeutics for treating human diseases [4, 5].

*Daphnia* are freshwater microcrustaceans that inhabit inland waters around the world and often are the critical herbivore in aquatic food webs [6, 7]. They have been a major model system in ecology, population genetics, and ecotoxicology for decades due to the ease with which field- and laboratory-based experiments can be conducted. Furthermore, they are cyclic pathenogens, a life cycle that permits genetically diverse natural populations and allows replication of genetically identical individuals through clonal reproduction in the lab [8]. With a fully sequenced genome (*D. pulex,* [9]), *Daphnia* has the potential to be a key model organism in molecular ecology and evolution, and is rapidly emerging as a model organism in non-ecological fields including biology of aging [10–15], and neurobiology [16–20]. Methodology for successful gene knockout using TALEN and CRISPER technologies exists for Daphnia [21, 22]. The U.S. National Institutes of Health list *Daphnia* as a model organism for biomedical research (http://www.nih.gov/science/models/) citing their extreme phenotypic responses to environmental changes, clonal reproduction, and ecological diversity as advantages in comparison to established biomedical models.

In order to fully realize *Daphnia* for molecular studies, techniques of experimental genetic manipulation are essential. Currently there is only a single technique described for RNAi in *Daphnia* [23, 24], which involves microinjecting small dsRNA molecules into the embryos of *D. pulex* and *D. magna*. Although this system has allowed for the study of some genes regulating embryonic development, there are several drawbacks and limitations to the microinjection method. The microinjection process is technically challenging, involves specialized equipment, thereby making the protocol expressive, tedious and unlikely to be broadly adopted by researchers interested in *Daphnia* [3, 25]. Currently, microinjection has been performed successfully only in *Daphnia* embryos. Therefore, this method limits the number of genes one can target as it is mainly applicable to genes expressed during embryonic development. Thus, there is no reliable RNAi method for studying genes expressed later in *Daphnia* life span, or for achieving knockdown for a specific duration during the life span.

In *C. elegans*, the problems associated with microinjection for RNAi were mitigated with the introduction of a new technique that involved feeding the worms with bacteria expressing specific dsRNAs [26–28]. This method for systemic RNAi via feeding has been adapted for multiple organisms including, but not limited to, the house cricket (*Acheta domesticus*), the lepidopteran pest *Spodoptera exigua*, the brown apple moth (*Epiphyas postwittana*), the termite *Reticulitermes flavipes*, and planarians [29–33]. Since the initial report of the systemic RNAi via feeding method in *C. elegans*, several different genes have been identified as being essential for systemic RNAi via feeding. One of these essential genes is Sid-1 (systemic RNAi defective), which encodes a transmembrane protein that forms a dsRNA gated channel [34–36]. We searched the recently published *Daphnia* genome [9] and found that it contains a Daphnid homologue of Sid-1. We also analyzed the *D. pulex* genome for the various proteins known to be involved in RNAi [1] using the online tool PANTHER (Protein Analysis Through Evolutionary Relationships) that allows for identification of various protein homologs based on domain structures and evolution of protein function in various organisms [37]. We determined that the *D. pulex* genome contains three homologs of Dicer, two homologs of Argonaute, and two homologs of TRBP [9]. Thus, we reasoned that if systemic RNAi via feeding could be achieved for *Daphnia*, transient gene knockdown experiments would rapidly advance development of this organism as a model system. In our present study, we present a method for efficient RNAi mediated gene knockdown in *Daphnia* via feeding.

Our study used three different species, *D. melanica, D. pulex, and D. pulicaria*. Although these taxa have different names, they are part of the same species-complex [38–44], hybridization among them is frequent in the wild [45–47], and experimental crosses do not exhibit reproductive isolation [48]. Therefore, they have limited divergence of their genomes, and can be used in complementary assays of genetic function. They were labeled as distinct species by molecular taxonomists based on mitochondrial divergence [38, 39], though they also have separate ecological niches, with *D. melanica* specialized for habitats with high UV radiation, and *D. pulex* and *D. pulicaria* specialized to small ponds and large lakes with low UV respectively [6, 42, 44, 49]. *D. melanica* produces melanin as a protective pigment for the high amounts of UV-radiation in its natural habitat [44, 50]. We used the melanin synthesis pathway of *D. melanica* to develop our RNAi technique, as it provides us with an easily measurable visible phenotype (loss of pigmentation) to assess the effectiveness of gene knockdown. We used *D. pulex* as a comparison because it is the closest relative to *D. melanica* that does not exhibit pigmentation [50].

We selected to target *phenoloxidase* gene*,* which encodes an enzyme essential for melanin synthesis in crustaceans, as a target for RNAi [51, 52] based on the easily identifiable phenotype it would produce if RNAi was successful. Our results provide evidence for establishment of an easy, feeding-based RNAi method in *Daphnia*. We demonstrate reduced *phenoloxidase* mRNA levels, diminished melanin levels resulting in a dramatic phenotype, and reduced survival in response to UV radiation in *Daphnia* that are fed on bacteria that express a dsRNA specific for *phenoloxidase*. This is the first demonstration that systemic RNAi is possible in *Daphnia.*

## Methods

### *Daphnia* cultures

*Daphnia pulex* was isolated from waterbodies in southwest Michigan in 2008 and have since been cultured in the lab. *Daphnia melanica* were isolated from high altitude alpine lakes in the Sierra Nevada region in eastern California. The isolate used was known as “Sierra”, and ND5 mitochondrial gene sequencing confirmed the *D. melanica* species identity [50, 53]. *D. pulex* species identity was determined by ldh allozyme characterization (designated “slow-slow”) as described previously [54, 55]. *D. pulex was* maintained at a temperature of 20° C with a photoperiod of 12:12 light:dark in a Percival growth chamber. *D. melanica* were maintained at a temperature of 15° C with a photoperiod of 16:8 light:dark. All *Daphnia* were maintained at a concentration of 3 to 5 animals per 250 ml beaker in 100 ml of filtered lake water until experimentation. Lake water was obtained from public access Lake Murray in central South Carolina and was filtered (1 μm) before use. Young newborn *Daphnia* were transferred to a new beaker with fresh water on alternate days. *D. pulex* cultures were fed every day with vitamin-supplemented algae *Ankistrodesmus falcatus* at a concentration of 20,000 cells/ml. *D. melanica* cultures were fed 20,000 cells/ml of *Ankistrodesmus falcatus* on alternate days.

### Vectors and feeding system

**Fig. 1:**
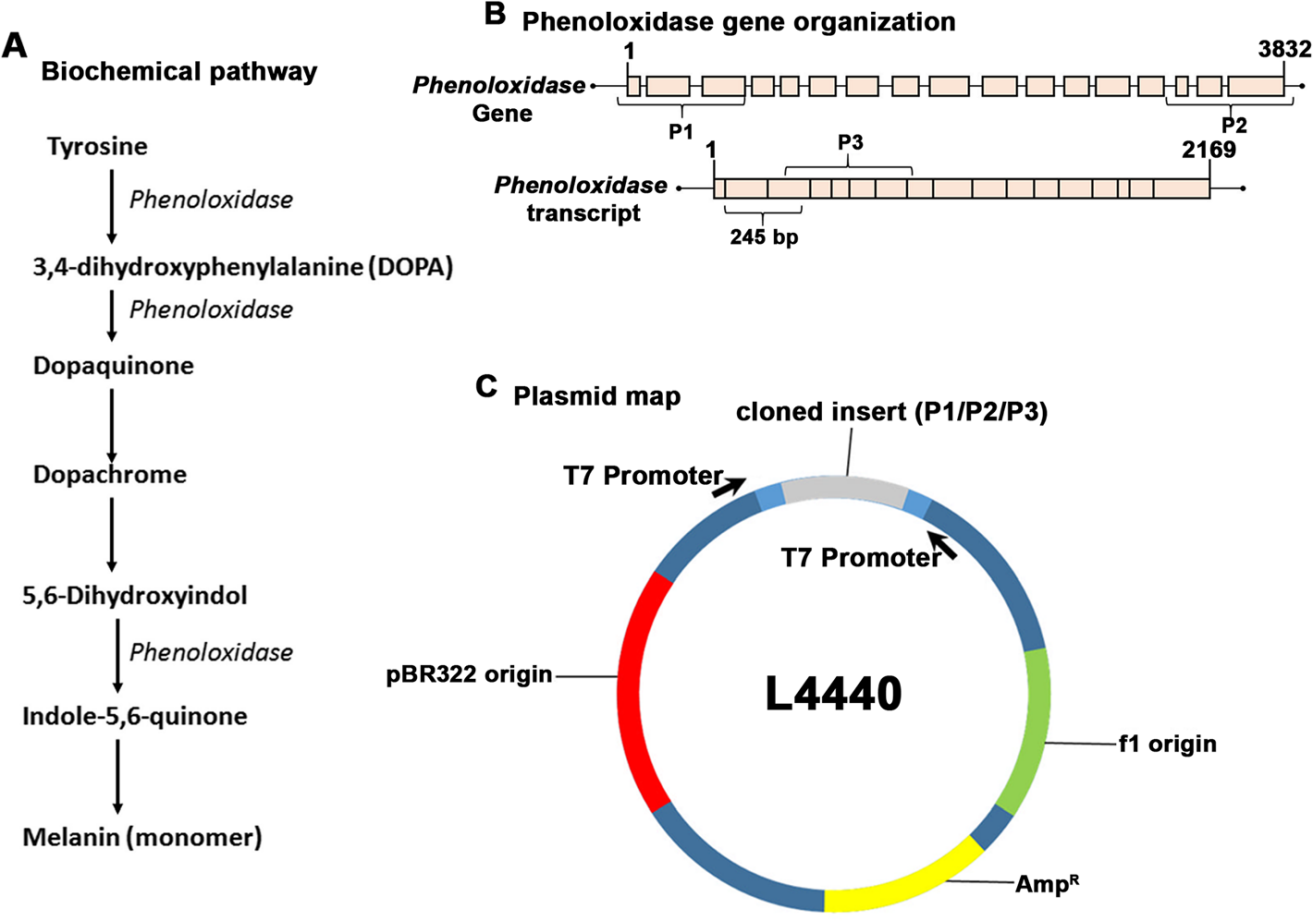
Arthropod Melanin Synthesis pathway and schematic map of regions selected for dsRNA expression constructs in *E. coli* plasmid L4440. **a** Arthropod melanin synthesis pathway. **b** *Daphnia phenoloxidase* gene and transcript. Marked on each diagram are the regions amplified using PCR and cloned in plasmid L4440. **c** Schematic diagram of the L4440 plasmid vector. L4440 is designed to produce double stranded RNA of the cloned insert between the two T7 promoters

We selected the L4440 plasmid vector for generating dsRNAs in an inducible manner in *E. coli*. L4440 plasmid vector (Fig. 1b) was designed by Fire et al. [56]. This vector allows cloning of PCR products between two T7 promoters in opposite orientations. The following two bacterial strains harboring λDE3 lysogen (a source of T7 RNA polymerase) were used for generating dsRNA from the recombinant plasmids:

1. BL21(DE3): ompT hsdS_B_ (r_B_-m_B_-) gal dcm (Novagen) - a strain deficient in lon and ompT proteases and used for efficient production of recombinant proteins.
2. HT115 (DE3) (W3110, rnc14::DTn10 (Addgene, [57]) - a strain deficient in RNase III and used for efficient production of dsRNAs.

*E. coli* cells that are (DE3) contain an integrated T7 RNA polymerase ORF under the control of a LacUV5 promoter the cultures can be induced with 2 mM IPTG to produce T7 RNA polymerase. This leads to the production of dsRNA from the PCR product cloned between the two T7 promoters. We also used the plasmid construct L4417, which contains the 5′ half (750 bp) of the GFP ORF cloned between the two T7 promoters (to be used as a negative control in the experiments as it does not have a natural target RNA in *Daphnia*). All the recombinant plasmids that we generated would produce a dsRNA product of about 800 bp in the bacterial host that expresses T7 RNA polymerase. Both L4440 and L4417 were obtained from Addgene (L4440 Plasmid #: 1654, L4417 Plasmid #1649- both gifts from Dr. A. Fire to Addgene).

### Target genes and plasmid constructs

In our current study, we targeted *phenoloxidase* transcripts for degradation by RNAi. The PCR primers were designed based on *D. pulex phenoloxidase* (NCBI_GNO_8300047) sequence. We generated several constructs using L4440 plasmid vector to generate dsRNA corresponding to different regions of our target transcripts (Fig. 1c and d). Note for each construct the PCR product was cloned into pGEMT Easy (Promega) and the sequence was confirmed. The following primers were used to generate the PCR products using either the genomic DNA or cDNA as a template. Appropriate restriction enzyme sites were engineered into the 5′ends of both PCR primers for sub-cloning from pGEMT Easy to L4440. Primers PO245 and GAPDH were used only in qPCR tests of gene expression levels.

**P1**: (gDNA template) Forward: CACCATGTCAGATTTGCAGC

Reverse: CGCAACATTTGCCTCTTACC

**P2:** (gDNA template) Forward: AATTCTTGCCGATCAAGGTG

Reverse: GCGAAATACGAACGAGGAAA

**P3**: (cDNA template) Forward: GCGTGGCAGGTTATTTTCAT

Reverse: CTTTAGAACGAGCCCAGACG

**PO245** (qPCR): Forward: CCATTCAGTCCTAAACCGGA

Reverse: ACCGTCGGAGCATTCTTAAA

**GFP:** Forward: GCCCGAAGGTTATGTACAGG

Reverse: AAAGGGCAGATTGTGTGGAC

**GAPDH:** Forward: TTATCACCTCCTCAACTTC

Reverse: CTTCTTCCTTCACTTCTCC

The following abbreviations denote which targeting vector was in the bacteria administered as feed to *Daphnia*: EV: Empty Vector L4440, GFP: GFP Control (L4417), P1: 5′ half of *phenoloxidase* gene amplified from genomic DNA, P2: 3′ half of *phenoloxidase* gene amplified from genomic DNA, P3: internal region of *phenoloxidase* mRNA amplified from cDNA, NM: Nonmelanic *D. pulex* (Clone: RW20), served as a control.

### RNAi feeding protocol

Ten *Daphnia* aged 4–5 weeks selected for experimentation were placed in 100 ml of filtered lakewater in a 250 ml beaker. *E. coli* strain BL21(DE3) bacteria transformed with the plasmid of choice (L4440 with one of the inserts indicated in Fig. 1) were grown overnight in Luria Broth (LB) with 2 mM IPTG to induce the expression of T7 RNA polymerase and the dsRNA corresponding to the cloned PCR product. The OD_600_ of the overnight cultures was measured, and bacteria from 2.8 OD_600_ units of overnight culture were pelleted (usually about 1 ml). The pelleted bacteria were resuspended in 1 ml of filtered lake water and dispensed directly into the beakers, which contained *Daphnia,* thereby diluting 1 ml of resuspended bacteria in 100 ml of filtered lake water. This corresponds to a final OD_600_ of 0.028 or about 2.4 × 10^7^*E. coli* cells in 100 ml. This same procedure was repeated for 10 days, with the *Daphnia* also being fed algae, *Ankistrodesmus falcatus,* at a concentration of 20,000 cells/ml each day for *D. pulex* and and on alternate days for *D. melanica*. The water being changed every other day for *D. melanica* or every day for *D. pulex*. New bacterial culture in fresh LB was prepared for feeding on each feeding day, and fresh algae (20,000 cells/ml) added after addition of bacteria to water, thus *Daphnia* were always fed with a mixture of algae and bacteria. During RNAi feeding regimen, the same photoperiods as stated under *Daphnia* cultures section were maintained for each species.

Initially we set up experiments to determine the optimal amount of bacteria and feeding duration for achieving effective RNAi. For this purpose, 10 *D. melanica* in 100 ml of lake water were fed on varying amounts of bacteria expressing the phenoloxidase dsRNA (P1) for 12 days. We recorded lethality as well as phenotypic response each day (Table 1) and the bacterial concentration that showed the best phenotype change and the least lethality was selected for analysis of phenoloxidase mRNA knockdown by Real Time PCR.

**Table 1:**
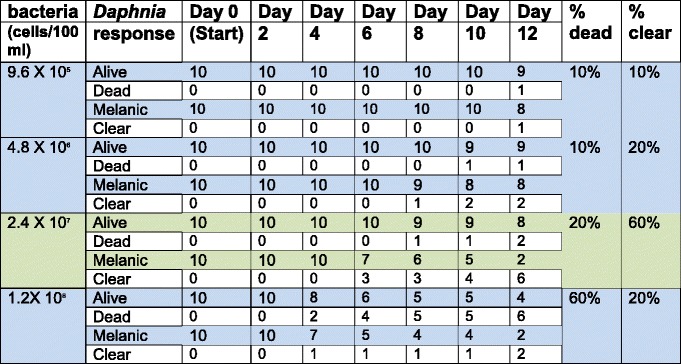
Optimization of the RNAi feeding regimen

We fed groups of 10 *D. melanica* in 100 ml of lake water on varying amounts of bacteria expressing the phenoloxidase dsRNA (P1) as indicated in the first column for a duration of 12 days. We recorded lethality (which is listed as the total number of *Daphnia* that had died from start of the experiment and overall % dead at the end of the experiment) as well as phenotypic response (which is listed as the total number of live *Daphnia* displaying a clear carapace on a particular day and overall % with clear carapace at the end of the experiment). The percentages were calculated based on starting number of individuals (10). Only the living Daphnia were scored for carapace color. The green rows and columns indicate the results representing the bacterial concentration that was selected for analysis of phenoloxidase mRNA levels on day 10. This was a concentration that exhibited the best phenotypic response

### UV treatment of *D. melanica*

For the experiments involving the knockdown of *phenoloxidase* in *D. melanica*, on the tenth and eleventh days of the bacterial feeding regimen, *Daphnia* were exposed to UV radiation by using a transilluminator with 312 nm UVB emission. *Daphnia* in 250 ml beakers (in 100 ml of water) were placed on the transilluminator. Beakers were arranged such that individual *Daphnia* were an average 10 cm from the UV source. Daphnia were exposed to UV for 5 min, and then returned to the Percival chamber. On the day 12 after beginning of bacterial feeding, the *Daphnia* were sacrificed and assayed for visual phenotypes and harvested to assay melanin content or isolate total RNA. This UV treatment allowed for a rigorous assessment of the RNAi targeting *phenoloxidase* via feeding method. *D. melanica*, if stressed, can stop production of melanin synthesis and this may result in false positive scoring of the loss of pigmentation phenotype since the presence of bacteria in water may induce some level of stress. By exposing *Daphnia* to UV radiation, the synthesis of melanin can be induced, over-riding any probable down regulation due to stress.

### Melanin assay

The assay was performed as per Hebert and Emery [58]. The body length of *Daphnia* to be analyzed via the Melanin Assay for their melanin content was measured and then *Daphnia* were placed in 50 μl of 5 M NaOH and incubated at 40 °C for 4 days. The melanin content of the resulting solution was determined by measuring optical density at 420 nm with a plate reader. We performed a melanin standard curve using commercially available bovine melanin. By generating a standard curve, we were able to convert our OD_420_ values into micrograms of melanin per millimeter of *Daphnia* [53, 58]. We also measured the melanin content of a nonmelanic *Daphnia* species as a negative control.

### UV sensitivity and survival assay

We tested *Daphnia’*s ability to survive UV exposure following the loss of pigmentation in response to feeding on bacteria expressing the *phenoloxidase* dsRNA. *Daphnia* in control and treatment groups were fed bacteria as described above for 10 days. On the eleventh day, *Daphnia* were subjected to UV radiation (10 min of UV radiation on the transillimuminator). We subjected the *Daphnia* a second time to this UV dosage on day 12 and on the thirteenth day examined the viability in control and treatment groups.

### Reverse transcriptase (RT)-PCR

Total RNA was isolated using RNAzol B reagent (TelTest) from 6 to 10 *Daphnia* following a bacterial RNAi feeding regimen for 10 days. Prior to RNA isolation, for the *Daphnia* fed on bacteria expressing any of the described dsRNAs, the entire gut was removed from each individual to avoid contamination from the bacteria in the gut that contain the dsRNA. *Daphnia* were collected in a microcentrifuge tube, rinsed once with 1 ml of PBS, and were homogenized in 0.8 ml of RNAzol B. Total RNA was isolated as per the supplied protocol. cDNA was synthesized using random hexamer primers, 1 μg total RNA, 10–20 units M-MuLV reverse transcriptase, 500 μM dNTPs, 40 units RNase Inhibitor RNasin (Promega) in appropriate reaction buffer. For each PCR reaction, 2 μl (1/10th of total) cDNA was used with 50 pmoles each of the forward and reverse primers designed to amplify *phenoloxidase* PCR product using the Promega GoTaq PCR kit. The following conditions were used for PCR: 95° C for 5 min (initial denaturation), denaturation at 95° C for 30 s, annealing at 58° C for 30 s, extension at 72° C for 30 s for 27 cycles in order to stay within linear range of amplification. The linear range was determined by varying cycle numbers and performing a densitometric analysis of the amplified product. PCR products were separated on a 1 % agarose gel.

### Real time PCR

We first determined the efficiency of the real time PCR reactions with serial dilutions of all cDNAs. Every reaction was performed in triplicate in a total volume of 20 μl. This included 4 μl cDNA, 250nM *phenoloxidase* or GAPDH primers, and SensiFast Supermix (BioLine). GAPDH was used for normalization. *Phenoloxidase* and GFP primers were validated by running serial dilutions with a template of known quality and the efficiency of the reactions were determined to be greater than 98 %. GAPDH primers were previously validated [53]. In addition, we also ensured that the GAPDH mRNA levels did not change during our treatments prior to performing experimental qRT-PCR for analyzing the phenoloxidase mRNA levels. In all samples analyzed (EV, GFP, P1, P2, P3 and *D. pulex*), the GAPDH threshold cycle (Ct) value was determined to be 24.5 +/− 0.10. This initial standardization was important because GAPDH mRNA levels were reported to change under certain treatment conditions in *Daphnia* [59]. All reactions were run on a BioRad CFX96 Real Time System C1000 Thermal cycler machine with the following conditions: 95° C for 30 s, 95° C for 5 s, 58° C for 5 s for *phenoloxidase* and 54 C for 5 s for GFP (the last three steps repeated for 50 cycles), 65° C for 5 s, and then 95° C for 5 s. We analyzed our data using the Bio-Rad CFX Manager Software with the 2^-ΔΔCt^ method. Note that three independent RNA isolations were used from three independent groups of *Daphnia* to serve as biological replicates.

### Statistics

To determine statistical significance, a two tailed Student’s *T*-test assuming equal variance or chi square analysis was performed. Each figure legend denotes p values as set forth by brackets and special F characters. Note that our alpha level was *p* = 0.05.

## Results and discussion

### Selection of target

In order to establish an RNAi method for *Daphnia* via feeding, we selected target genes that would result in easily identifiable visible phenotypes. A deficiency of phenoloxidase enzyme would result in a reduction of melanin pigment, thus producing a visible loss of pigmentation in *D. melanica*. As shown in Fig. 1a, we selected to target *phenoloxidase* gene based on its involvement in several essential steps in the melanin synthesis pathway [52, 60]. In order to test dsRNAs corresponding to three different regions of *phenoloxidase* gene for their effectiveness, we used three PCR primer pairs to amplify the indicated regions for sub-cloning into the plasmid vector L4440. As is shown in Fig. 1b, two regions from the *phenoloxidase* gene (with primers binding in introns of the gene) corresponding to the 5′ region (P1) and 3′ region (P2) were selected for PCR amplification. Another primer pair was used with cDNA as a template (therefore no intronic regions would be present), which corresponds to a central region of the *phenoloxidase* transcript (named P3, Fig. 1b). A different primer pair was used for real time PCR in order to measure changes in *phenoloxidase* transcript levels (termed PO245, Fig. 1b) after RNAi feeding regimen.

### Optimization of the RNAi feeding protocol

We selected the concentration of bacteria in feed as well as the duration of the RNAi feeding regimen based on our initial experiments to determine the effective dose that would result in the least lethality and a quick phenotypic response (loss of pigmentation in *D. melanica*). As seen in Table 1, we selected the bacterial dose that resulted in a phenotypic change in 10 days with the least lethality (which was 2.4 x 10^7^ bacterial cells/day/100 ml lake water for 10 days). Note that although this concentration and duration was optimal for targeting phenoloxidase in *D. melanica*, the optimal concentration and duration may have to be determined for specific species and clones of *Daphnia* as well as for different target genes using the RNAi via feeding system. The method used for this initial optimization would be similar to one we outline here.

### GFP dsRNA can be detected in *Daphnia* after being fed on bacteria expressing GFP dsRNA

**Fig. 2:**
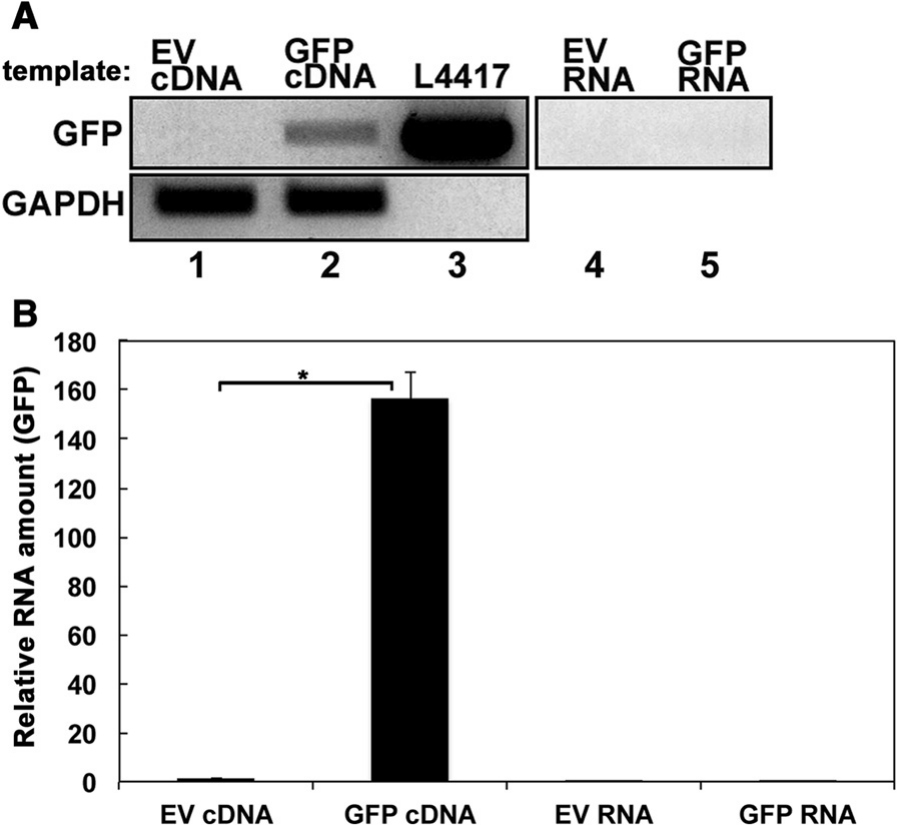
dsRNA generated in *E. coli* can be detected in *Daphnia*. **a** Reverse transcriptase-PCR data. Lane 1: RNA isolated from *Daphnia* fed on bacteria containing L4440 without any insert (empty vector-EV), Lane 2: RNA isolated from *Daphnia* fed on bacteria containing GFP/L4440, Lane 3: GFP/L4440 plasmid DNA (template positive control), Lanes 4 and 5: reverse transcriptase negative control (the negative control without reverse transcriptase ensures lack of plasmid DNA contamination). GAPDH was used as an internal control for ascertaining that equal amounts of *Daphnia* mRNA were analyzed in lanes 1 and 2. **b** Quantitative real time PCR data. Real Time PCR was performed with the same samples as in panel A. *P*-value: * = 5 × 10^−5^

First, we wanted to determine if the dsRNA expressed in bacteria was being effectively delivered to *Daphnia* after being fed on the bacterial suspension. To analyze this, we fed *D. melanica* bacteria expressing GFP dsRNA. The plasmid L4417 (GFP insert cloned in L4440) produces GFP dsRNA from two complementary RNA strands generated from two T7 promoters. Since GFP is not an endogenous *Daphnia* gene, the presence of GFP dsRNA in *Daphnia* would indicate that the dsRNA was delivered from bacteria to *Daphnia* gut and then to the rest of the body. To test this, RNA isolated from *Daphnia* fed on L4417 containing bacteria was subjected to reverse transcriptase PCR analysis. Before isolating total RNA for analysis, the guts of all *Daphnia* were removed carefully to ensure that RNA samples were not contaminated with bacteria from the feed. As is shown in Fig. 2a, GFP dsRNA was detected in *Daphnia* fed on bacteria with L4417 plasmid (lane 2) but not in *Daphnia* fed on L4440 (EV, lane 1). L4417 plasmid DNA was used as a template positive control for PCR and generated the expected PCR product (lane 3). In order to ensure that there was no plasmid DNA contamination in the RNA preparation originating from bacteria in *Daphnia* guts, we performed PCR using the isolated RNA (and not cDNA) without the reverse transcriptase step as a template. As seen in lanes 4 and 5, no PCR product was generated in the absence of reverse transcriptase reaction thereby confirming that the PCR product was being generated only from GFP dsRNA (Note: all RNA was treated with DNase before being analyzed). Fig. 2b shows a quantitative reverse transcriptase PCR (qRT-PCR) confirming our results from Fig. 2a. The L4417 template positive control was excluded from Fig. 2b because as expected it showed an extremely high level of amplification and PCR product. Thus we were able to detect non-endogenous GFP dsRNA in *Daphnia* fed on bacteria containing the plasmid L4417, demonstrating an effective delivery of dsRNA to *Daphnia* tissues other than gut. The results conclusively prove that GFP dsRNA is detected in *Daphnia* tissues other than the gut and indicates effective delivery of dsRNA.

### Phenotypic change in *D. melanica* fed on bacteria expressing phenoloxidase dsRNA

**Fig. 3:**
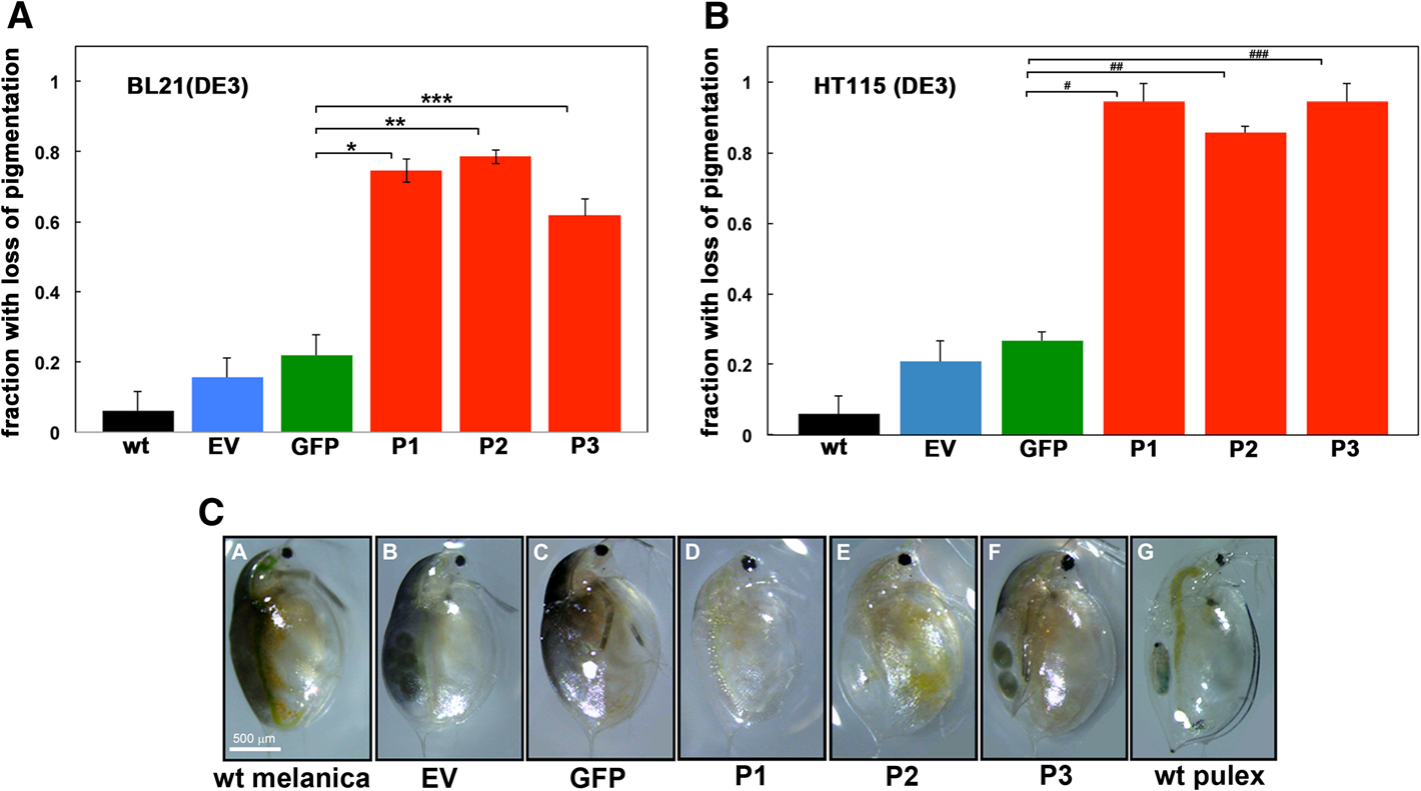
*Daphnia* fed on bacteria expressing the *phenoloxidase* dsRNA demonstrate a loss in melanin pigmentation. **a** and **b** Proportion of *Daphnia* demonstrating pigmentation loss phenotype after 10 days on an RNAi feeding regimen with BL21(DE3) and HT115(DE3) strain respectively. Following the feeding regimen, *Daphnia* were UV treated to induce melanin production and then observed under a dissecting scope for pigmentation phenotype. wt *(melanica)*: *n* = 50, EV (L4440) *n* = 51, GFP (L44417): *n* = 50, P1 (P1/L4440): *n* = 55, P2 (P2/L4440): *n* = 56, P3 (P3/L4440): *n* = 57. These abbreviations denote which plasmid was transformed into the bacteria before administering as feed to *Daphnia*. The data represent five replicate experiments. *P*-values are as follows: * = 0.0005, ** = 0.0032, *** = 0.0002, # = 1.2 × 10^−7^, ## = 2.0 × 10^−7^, and ### = 0.00016. **c** Photographic representation of *Daphnia* from each group as indicated below each panel. The non-melanic species *D. pulex* is shown for comparison

To investigate the effectiveness of RNAi via feeding method, we fed *D. melanica* on bacteria expressing the *phenoloxidase* dsRNA to target this essential enzyme in melanin biosynthesis pathway. After the RNAi feeding regimen, *Daphnia* were observed for loss of pigmentation and the percentages of *Daphnia* exhibiting loss of pigmentation were calculated. *Daphnia* were grouped either as pigmented or not pigmented. There were some variations in pigmentation loss phenotype and *Daphnia* showing greater than 50 % loss of pigmentation along the back of the carapace, between the gut and the edge of the carapace, were classified as loss of pigmentation while the ones showing less than 50 % loss were classified as pigmented. We performed this classification by analyzing photos of *Daphnia* each day of their RNAi feeding regimen. The majority of organisms that showed loss of pigmentation had completely clear carapace with no pigmentation. However, about 20 % of the ones classified as lacking pigmentation had partially clear carapaces with pigmentation apparent in 50 % or less carapace area. Demonstrated in Fig. 3, *D. melanica* fed on bacteria expressing *phenoloxidase* dsRNA displayed a remarkable loss of pigmentation (Fig. 3a and b). This phenotype was not seen in *D. melanica* that were fed L4440 plasmid vector containing no inserts (EV) or a plasmid vector expressing GFP dsRNA (L4417). No visible effect of the *phenoloxidase* dsRNA produced in bacteria was observed in nonmelanic *Daphnia* (*Daphnia pulex*, Clone: RW20) when fed on dsRNA producing bacteria. As is shown in Fig. 3a and b, *D. melanica* with no treatments showed a slight change in phenotype (Black bars, Fig. 3a and b, 6.0 +/− 5.2 %), which can be thought as natural variation in pigmentation. *D. melanica* fed on bacteria with EV (blue bars) and GFP control constructs (green bars) showed a pigmentation loss phenotype in some individuals (15.6 +/− 5.9 % and 22 +/− 5.4 % respectively) in comparison to the group with no treatment. *D. melanica* can show reduced melanin synthesis if they become stressed [61]. It is possible that the presence of any dsRNA (a short dsRNA may be produced from the polylinker in EV) could trigger stress signaling and elicit down regulation of melanin production and a minor but noticeable phenotype in a small percentage of individuals. However, *D. melanica* fed on bacteria expressing *phenoloxidase* dsRNA displayed a markedly high percentage of individuals with the pigmentation loss phenotype (Red bars, Fig. 3a and b) that was 4–6x greater than the non-target controls. In case of the BL21(DE3) bacterial strain, P1 displayed about 75 % (*n* = 55), P2 displayed 78 % (*n* = 56) and P3 was slightly more variable with about 62 % (*n* = 47) of *Daphnia* displaying the pigmentation loss phenotype (Fig. 3a). The bacterial strain HT115(DE3) that lacks the RNase III activity exhibited more efficient RNAi (Fig. 3b) as compared to BL21(DE3) strain (Fig. 3a) with P1 displaying about 94 % (*n* = 16), P2 about 86 % (*n* = 14) and P3 about 94 % (*n* = 16) of *Daphnia* with loss of pigmentation. Figure 3c shows a representative individual from each group of *Daphnia;* either untreated wild type, controls (fed on bacteria harboring EV L4440 or L4417) or fed on bacteria expressing *phenoloxidase* dsRNA (P1, P2, and P3). The dramatic loss of pigmentation is clearly evident in P1, P2, and P3 groups (panels D, E, and F) as compared to untreated and control samples (panels A, B, and C). Panel G shows a non-melanic *D. pulex* that was fed only on algae (20,000cells/ml) for comparison. These results indicate that RNAi method is successful and it is possible to achieve systemic RNAi via feeding in *Daphnia*. The bacterial strain HT115(DE3) was more effective in generating the RNAi phenotypes as expected since it is deficient in RNAse III and is known to accumulate dsRNA at high levels compared to BL21(DE3) strain. Although both BL21(DE3) and HT115(DE3) strains work in *Daphnia* to produce RNAi phenotypes, we recommend using HT115(DE3) strain for future experiments.

During the course of the phenoloxidase RNAi feeding regimen, some progeny organisms born to treated mothers (about 10–15 %) exhibited clear carapaces, while others in the same clutch were melanic. Due to the unpredictability of the clear offspring being born over the course of the experiment, we did not analyze steady state levels of phenoloxidase mRNA in the progeny with clear carapaces. Once removed from the RNAi feeding regimen, the offspring with clear carapaces always returned to normal melanic pigmentation within the next 4–6 days. It is worth a note that the survival as well as the total offspring number was not affected by the RNAi feeding regimen.

### Phenoloxidase transcript levels are diminished in *Daphnia* fed on bacteria expressing Phenoloxidase dsRNA

**Fig. 4:**
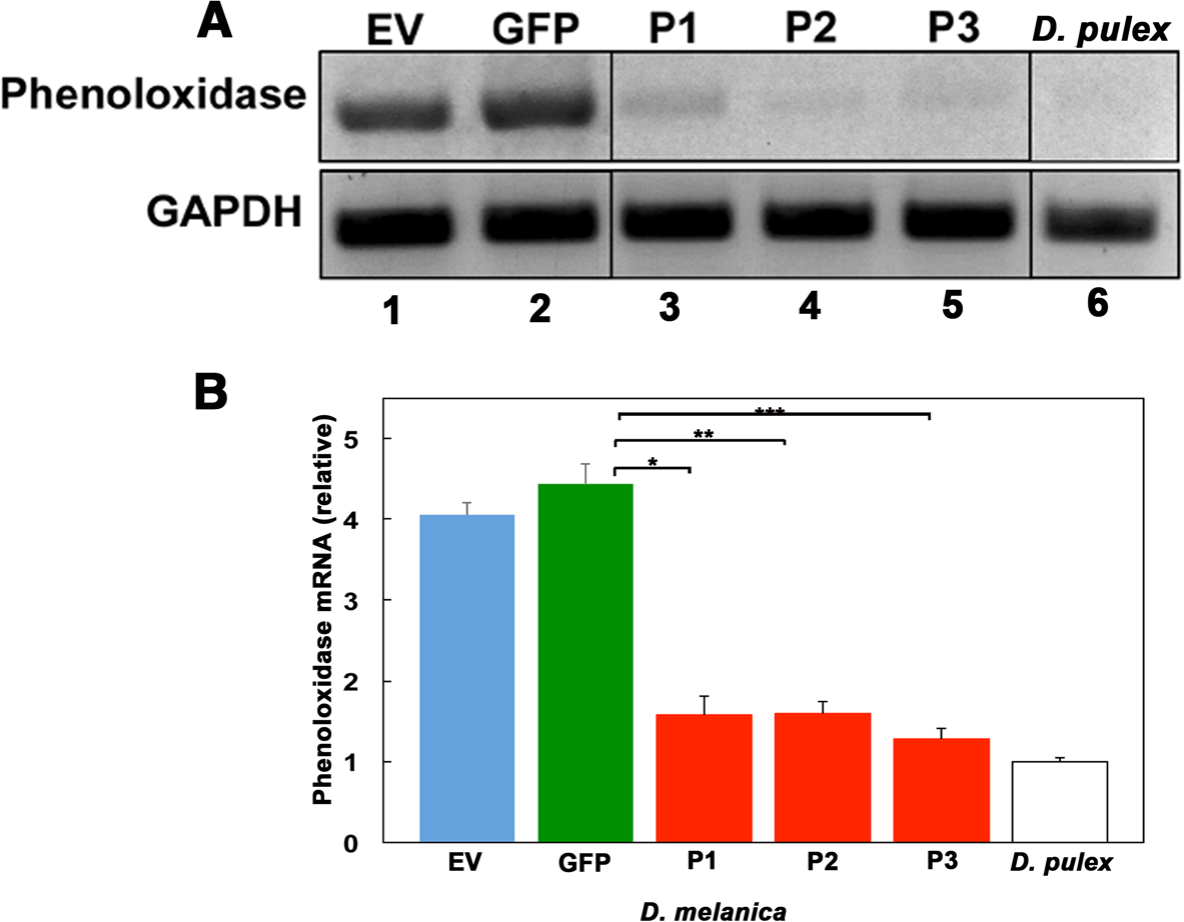
*Phenoloxidase* transcript levels are diminished in *Daphnia* fed on bacteria that express *phenoloxidase* dsRNA. **a** Reverse Transcriptase PCR examining the expression of *phenoloxidase* mRNA. RNA samples are as follows- Lane 1: EV (L4440 without any insert), Lane 2: GFP (GFP/L4440), Lane 3: P1 (P1/L4440), Lanes 4: P2 (P2/L4440), Lane 5: P3 (P3/L4440), and Lane 6: non-melanic *D. pulex* (isolate RW20) control. GAPDH was used as an internal control for ascertaining that equal amounts of *Daphnia* mRNA were analyzed in each lane. **b** Quantitative real time PCR examining the expression of *phenoloxidase* mRNA. The abbreviations under each bar denote which plasmid vector was transformed into the bacteria before administering as feed to *Daphnia. P*-values are as follows: * = 1 × 10^−7^, ** = 7.2 × 10^-8,^ and *** = 9.8 × 10^−8^. Blue bar: EV, Green bar: GFP, red bars: P1, P2, and P3, white bar: non-melanic *D. pulex* control

Since there was an obvious phenotypic change in *D. melanica* fed on bacteria expressing *phenoloxidase* dsRNA, we analyzed the *phenoloxidase* mRNA levels by reverse transcriptase (RT)-PCR (Fig. 4a) as well as quantitative Real Time (qRT)-PCR (Fig. 4b). As is shown in Fig. 4a, *phenoloxidas*e mRNA levels are markedly diminished in *Daphnia* fed on bacteria expressing *phenoloxidase* dsRNA (lanes 3–5) as compared to controls (lanes 1–2). Comparing lanes 1 and 2, it appears that there is more *phenoloxidase* mRNA in the GFP fed *Daphnia*. The presence of dsRNA may elicit an immune response and phenoloxidase is also a key enzyme in responding to immune insults in *Daphnia* [52]. Nevertheless, all the *phenoloxidase* targeting constructs (including those with regions corresponding to genomic DNA) resulted in diminished levels of *phenoloxidase* mRNA (lanes 3–5). We also used untreated, non-melanic *D. pulex* (RW20) as a negative control for PCR (lane 6, Fig. 4a). Shown in Fig. 4a and b, *D. melanica* fed on bacteria expressing *phenoloxidase* dsRNA displayed levels of *phenoloxidase* mRNA similar to non-melanic *Daphnia* (RW20). Thus, when *Daphnia* are fed on bacteria expressing *phenoloxidase* dsRNA, the *phenoloxidase* mRNA levels decline significantly, thereby demonstrating achievement of very effective RNAi.

### Melanin levels are diminished in *Daphnia* fed on bacteria expressing phenoloxidase dsRNA

**Fig. 5:**
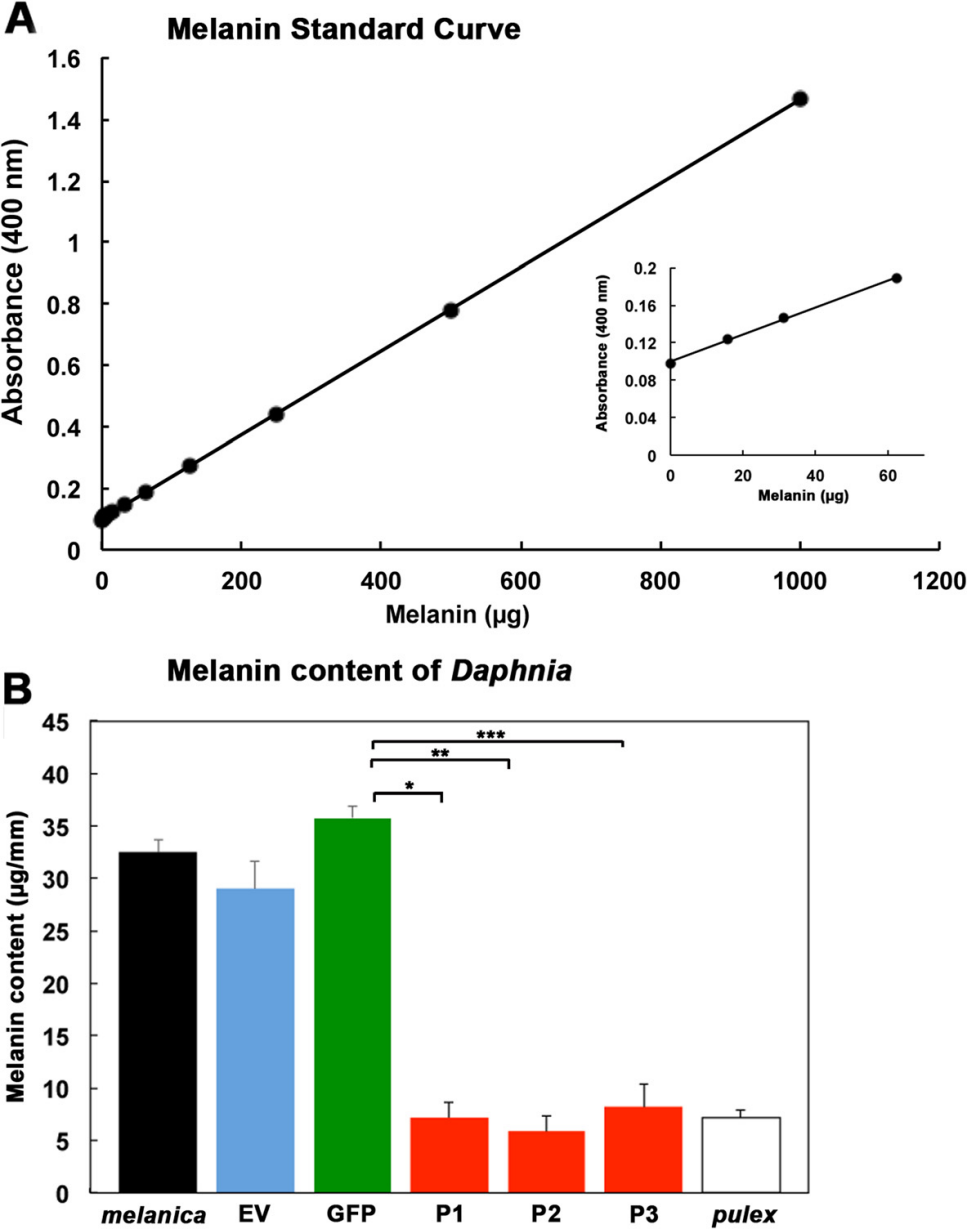
Melanin content is diminished in *D. melanica* fed on bacteria expressing *phenoloxidase* dsRNA. **a** Melanin standard curve using purified melanin (Sigma). Melanin was dissolved in 5 M NaOH before making serial dilutions to establish a standard curve. **b** Melanin Assay performed using extract from various treated *Daphnia*. wt *D. melanica* (*melanica*): *n* = 14, EV: *n* = 15, GFP (GFP/L4440): *n* = 14, P1 (P1/L4440): *n* = 11, P2 (P2/L4440): *n* = 12, P3 (P3/L4440): *n* = 13, D. pulex RW20 (*pulex)*: *n* = 20. Data represents 2 replicate experiments. The abbreviations indicated below the bars denote the plasmid vector that was transformed into the bacteria before administering as feed to *Daphnia,* except for *pulex*, which indicates non-melanic control without bacterial feed. *P-* values are as follows: * = 0.001, ** = 0.0002, and *** = 0.0016

We further validated that RNAi was achieved by measuring melanin content of *Daphnia* to ensure that the observed phenotypic changes were due to a decline in melanin. The melanin content was determined in *Daphnia* fed on control bacteria (Fig. 5b, EV and GFP) and *Daphnia* fed on bacteria expressing the *phenoloxidase* dsRNA (Fig. 5b, P1, P2, and P3). First we established a standard absorbance curve for various melanin concentrations using commercially available melanin (Fig. 5a). Using the standard curve, the melanin content of *Daphnia* in experimental groups was determined and is shown in Fig. 5b. Wild type *D. melanica* (black bar), and the control organisms (blue bar: EV, and green bar: GFP) contained significantly higher amounts of melanin (32.49 +/− 1.27 μg/mm, 29 +/− 2.5 μg/mm, and 35.8 +/− 1.06 μg/mm respectively) than *Daphnia* fed on bacteria expressing *phenoloxidase* dsRNA (red bars: P1: 7.2 +/− 1.48 μg/mm, P2: 5.81 +/− 1.5 μg/mm, and P3: 8.21 +/− 2.16 μg/mm of melanin). We included nonmelanic *D. pulex* as a negative control (white bar: 7.147 +/− 0.75 μg/mm of melanin). Comparing P1, P2, and P3 to the non-melanic *Daphnia*, the amount of melanin present is very similar, showing that feeding on bacteria expressing *phenoloxidase* dsRNA resulted in a marked reduction in melanin levels to bring them to the levels in non-melanic *D. pulex* (Fig. 5b). Thus, the diminished levels of *phenoloxidase* mRNA results in a corresponding decrease in melanin content in *Daphnia*.

### *Daphnia* fed on bacteria expressing phenoloxidase dsRNA are sensitized to UV radiation

**Fig. 6:**
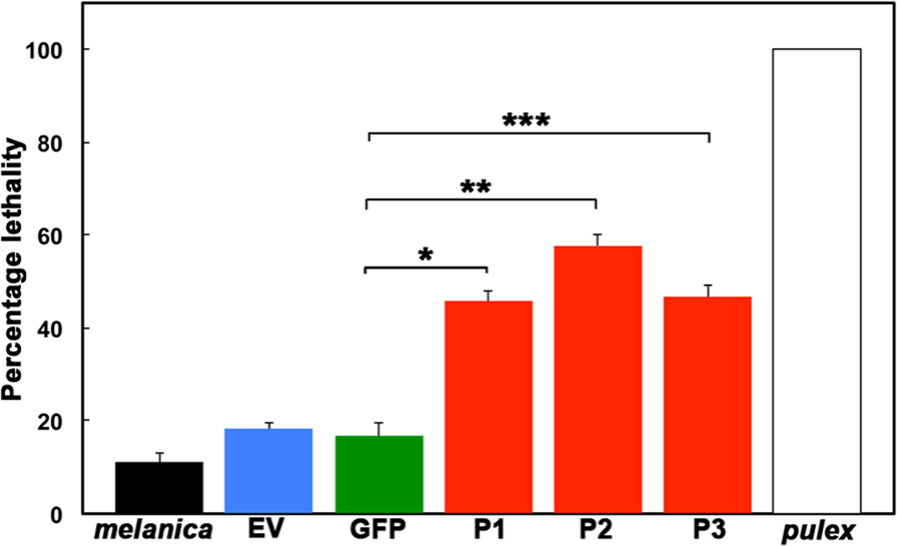
Enhanced UV sensitivity of *D. melanica* fed on bacteria expressing *phenoloxidase* dsRNA**.** *D. melanica* were subjected to the RNAi feeding regimen for 10 days and then exposed to UV each day for 2 consecutive days. The percent viability of *Daphnia* was assessed 24 h after the second exposure. *D. pulex* was used as a control and all individuals died within 2 h of the initial UV insult. wt *melanica* (*melanica*): *n* = 18, EV: *n* = 22, GFP (GFP/L4440): *n* = 24, P1 (P1/L4440): *n* = 24, P2 (P2/L4440): *n* = 26, P3 (P3/L4440): *n* = 30, *D. pulex* RW20 (*pulex*): *n* = 43. These abbreviations denote the plasmid that was transformed into the bacteria before administering them as feed to *Daphnia.* Data represent 3 replicate experiments. *P*-values are as follows: * = 0.0293, ** = 0.0028, and *** = 0.0201

To investigate the functional effect of melanin loss, *Daphnia* were exposed to UV radiation following the loss of pigmentation after an RNAi feeding regimen. Since melanin protects the DNA damage in response to UV radiation [62], we predicted that the *Daphnia* exhibiting loss of pigmentation in response to RNAi knockdown of *phenoloxidase* would be more sensitive to UV. For *D. melanica*, melanin pigmentation in the wild helps them cope with large amounts of UV radiation they encounter daily [44, 50]. *Daphnia* are able to induce the production of melanin following exposure to UV light [50, 53]. This is particularly important for organisms inhabiting regions high in altitude because their UV exposure tends to be high [44, 50]. The production of melanin is a protective measure, mitigating the DNA damage resulting from of high doses of UV [50]. Thus, *Daphnia* were treated with UV radiation and their viability was measured following UV exposure. As shown in Fig. 6, *Daphnia* that were fed on bacteria expressing *phenoloxidase* dsRNA displayed a dramatically increased sensitivity to UV with about 50 % lethality (red bars: P1: 45.8 +/− 2.9 %, P2: 57.7 +/− 3.2 %, and P3: 46.7 +/− 3.5 % death). In contrast to this, wt *D. melanica* (black bar) and those fed on bacteria with EV (blue bar) or L4417 GFP control (green bar) showed markedly less lethality (EV: 18.1 +/− 1.4 %, GFP: 16.7 +/− 3.92 %). The non-melanic *D. pulex* was used as a negative control (white bar) and following UV radiation all *D. pulex* individuals died within 2 h (*n* = 43). These results further confirm that RNAi targeting of *phenoloxidase* worked efficiently in *Daphnia* and exhibited markedly reduced *phenoloxidase* mRNA, melanin content, and markedly increased UV sensitivity. Thus, the knockdown of *phenoloxidase* transcript in *D. melanica* results in a significant reduction in the ability to survive after UV exposure, further validating our RNAi method.

### Delivery of dsRNA by feeding is a convenient and reliable RNAi method

In order to knock down specific gene expression, several methods for the delivery of dsRNA or siRNA have been used in recent years. Among these, RNAi via direct injection of a dsRNA solution is a simple method that works in larger insects due to the easy protocol. It is effective in knocking down expression of target genes in invertebrates such as the cricket *G. bimaculatus*, the mosquito *Aedes aegypti* [63], the German cockroach *Blattella germanica* [64], and the silkworm larvae *Bombyx mori* [65]. For a small freshwater microcrustacean such as *Daphnia*, it is difficult to achieve RNAi via injection in adult organisms as injection may result in high mortality since *Daphnia* will lose viability rapidly if not kept immersed in cool water. Thus, delivering dsRNA by feeding offers several advantages over direct injection of dsRNA method, as it is less labor-intensive, less expensive, and is also applicable for screening a large number of essential *Daphnia* genes because of its simplicity. Our study also suggests for the first time that RNAi in *Daphnia* is systemic, and is the first report of *phenoloxidase* knockdown using RNAi which not only produced significant reduction in mRNA and melanin levels but it also resulted in a marked increase in lethality in response to UV exposure.

It is worth noting that melanin synthesis was restored in *D. melanica* at about 7 days after bacterial feeding was discontinued, thereby demonstrating that the RNAi was transient and thus this method holds a potential to test the effect of gene knockdown at selected time points during the life span. This may be a very useful feature, especially for aging and longevity research to study the contribution of specific genes in an age-dependent manner. As there was no apparent effect of RNAi bacterial feeding regimen on reproduction in any species we examined, the technique would be widely applicable to most genes. In this regard, it is worth noting that we attempted a knockdown of *distal-less* and *eyeless* by the maternal feeding method. A knockdown of distal-less produced no viable progeny, which could be a sign of embryonic lethality specifically due to achieving efficient RNAi for *distal-less. Drosophila distal-less* null mutants die as embryos due to defects in development of sensory organs [66]. The knockdown of eyeless was effective and produced a deformed eye phenotype in the progeny as early as in the first clutch after 4–5 days of RNAi feeding regimen. However the organisms exhibiting deformed eyes did not survive for more than a few hours and no RNA could be isolated for analysis of eyeless mRNA levels. Thus, although the preliminary data suggests that targeting mRNAs expressed in developing embryos via feeding the mothers is possible, further experiments are essential to establish the experimental conditions and validation of RNAi effects.

Thus, we present a fast and effective method to achieve gene-specific knockdown in adult organisms as well as developing *Daphnia* embryos that holds a tremendous potential to become a mainstream method in various types of biological studies that use *Daphnia* as a model organism.

## Conclusions

We describe a new method to achieve gene specific knockdown by RNAi in *Daphnia* via feeding. By using *E. coli* cells that express gene-specific dsRNAs as a food additive for adult *Daphnia*, we can achieve an efficient RNAi for genes that are expressed in adult tissues. This method provides a powerful tool for genetic manipulation of this important model organism for environmental, evolutionary, as well as developmental genomics.
